# Language influences on numerical development—Inversion effects on multi-digit number processing

**DOI:** 10.3389/fpsyg.2013.00480

**Published:** 2013-08-05

**Authors:** E. Klein, J. Bahnmueller, A. Mann, S. Pixner, L. Kaufmann, H.-C. Nuerk, K. Moeller

**Affiliations:** ^1^Section Neuropsychology, Department of Neurology, University Hospital RWTH Aachen UniversityTuebingen, Germany; ^2^IWM-KMRC Knowledge Media Research CenterTuebingen, Germany; ^3^Department of Psychology, Eberhard Karls UniversityTuebingen, Germany; ^4^Department of Psychology, UMIT – The Health and Life Sciences UniversityHall/Tyrol, Austria; ^5^Department of Psychiatry and Psychotherapy A, General HospitalHall in Tyrol, Austria

**Keywords:** number processing, numerical development, multi-digit number comparison, inversion effects, language-moderated effects

## Abstract

In early numerical development, children have to become familiar with the Arabic number system and its place-value structure. The present review summarizes and discusses evidence for language influences on the acquisition of the highly transparent structuring principles of digital-Arabic digits by means of its moderation through the transparency of the respective language's number word system. In particular, the so-called inversion property (i.e., 24 named as “four and twenty” instead of “twenty four”) was found to influence number processing in children not only in verbal but also in non-verbal numerical tasks. Additionally, there is first evidence suggesting that inversion-related difficulties may influence numerical processing longitudinally. Generally, language-specific influences in children's numerical development are most pronounced for multi-digit numbers. Yet, there is currently only one study on three-digit number processing for German-speaking children. A direct comparison of additional new data from Italian-speaking children further corroborates the assumption that language impacts on cognitive (number) processing as inversion-related interference was found most pronounced for German-speaking children. In sum, we conclude that numerical development may not be language-specific but seems to be moderated by language.

The Arabic number system is the world's most widely-used number system (see Zhang and Norman, 1995; Chrisomalis, 2004; Widom and Schlimm, 2012, for taxonomies of number systems). It relies on a simple formal structure: Based on a set of ten symbols (i.e., 0, 1, 2, 3, 4, 5, 6, 7, 8, 9) it is possible to assemble any possible number. All one has to consider is its *place-value structuring principle* which defines that the overall magnitude of a multi-digit number is coded by its constituting digits organized in descending powers of the basis 10 from left to right (i.e., 316 = {3} ×10^2^ + {1} ×10^1^ + {6} ×10^0^). So, this principle defines the numerical value of each individual digit in a multi-digit number in digital-Arabic notation by its position within the respective digit string.

In early numerical development, children have to become familiar with Arabic numbers in general and, in particular, they have to understand the place-value principle with its underlying base-10-structure. However, this world-wide uniform and transparent combination principle only applies to numbers in digital-Arabic notation. In contrast, verbal number word systems differ between languages in the way they correspond to the systematic and language-independent place-value structuring of digital-Arabic numbers. This is often referred to as the transparency of a number word system, describing how closely a language's number word system conforms to the structure of a digital number system (Dowker et al., 2008). Importantly, there is accumulating evidence that difficulties in understanding the Arabic number system and interferences on number processing are associated with the transparency of number word systems (e.g., Seron and Fayol, 1994; Nuerk et al., 2005a; Zuber et al., 2009; Pixner et al., 2011a). In particular, language (by means of its number word structure) moderates multi-digit number processing as indicated by a variety of language moderated effects in adults but also in children (see Nuerk et al., 2011 for a review). However, the question when and how these language modulations become important in numerical development is still not answered sufficiently.

Several studies showed that children speaking a language with transparent number words have fewer problems acquiring basic arithmetical competencies. In particular, most Asian languages (e.g., Japanese, Korean, Chinese) have a transparent number word system (Comrie, 2005). For instance, in Japanese 452 is literally coded as “four-hundred-five-ten-two” (“yon-hyaku-go-jū-ni”). In this vein, Miura et al. (1993) showed that Asian as compared to English-speaking first graders exhibited better understanding of the place-value structure of the Arabic number system when asked to, e.g., explicitly identify tens and units of a two-digit number. Based on such results, it has also been argued that the higher mathematical achievement observed repeatedly for Asian as compared to European and American children may benefit from their more transparent number word systems (Miura et al., 1993, 1994; Towse and Saxton, 1998). This argument was corroborated by a recent systematic review by Ng and Rao (2010) indicating that these differences in mathematical achievement cannot be accounted for entirely by cultural influences (i.e., educational system, student motivation, etc.) but are—at least in early mathematical development—driven by the benefits of Asian number word systems.

This seems plausible when considering that the number word systems of most European languages are less transparent. For instance, there are specific words for multiples of ten (e.g., “forty” and not “four ten”), teen numbers (e.g., “sixteen” and not “ten six”), number words with a base other than ten (e.g., in French 82 is named as “quatre-vingt-deux” which translates to “four twenty two”). One of the most important inconsistencies in number words common to several languages (e.g., Arabic, Danish, German, Maltese, etc.) is the inversion property. In this context, inversion describes the fact that the order in which tens and units are named in number words is inverted compared to their order in digital-Arabic notation. For instance, in German 24 is named “vierundzwanzig” which literally translates to “fourandtwenty.” Mastering this inconsistency poses one of the most common challenges in early numerical development for children speaking a language with inversion. Importantly, difficulties related to the inversion property are not restricted to transcoding and the use of number words but also generalize to the processing of number magnitude.

## Inversion effects on transcoding

Influences of the inversion property on verbal numerical tasks such as transcoding have been shown repeatedly. For instance, Krinzinger et al. (2011) evaluated transcoding performance of 2nd graders from France, Wallonia, Flanders, Germany, and Austria. Their results indicated inversion-based between-language effects when pupils had to write down Arabic numbers to dictation: those speaking a language with inverted number words (i.e., Flemish, Austrian, German) made generally more transcoding errors as those speaking a language without inversion. More particularly, Zuber et al. (2009) investigated inversion-related transcoding errors in German-speaking 1st graders and observed that almost half of their errors were related to the inversion property of German number words. In line with this, Nuerk et al. (2005a) observed that German-speaking 1st graders not only committed significantly more transcoding errors in general as compared to Japanese-speaking children but more inversion-related errors in particular.

Interestingly, there are two number-word systems in Czech—one with and one without inversion. Pixner et al. (2011b) observed that the same children committed more errors and, in particular, more inversion-related errors when asked to transcode number words dictated in their inverted compared to their non-inverted form. These results and the fact that inversion-related transcoding errors have not been reported for languages without inversion (French: Barrouillet et al., 2004; Camos, 2008; Italian: Power and Dal Martello, 1990, 1997) clearly suggest that intransparencies of a language's number word system such as the inversion property may impede the acquisition of basic numerical skills.

## Inversion effects on the processing of number magnitude

Influences of inversion have also been observed for tasks with no explicit involvement of number words such as Arabic number magnitude comparison and number line estimation. As regards magnitude comparison, the so-called unit-decade compatibility effect (UDCE, Nuerk et al., 2001) was found to be moderated by inversion. The UDCE describes the finding that magnitude comparisons of unit-decade-compatible number pairs (e.g., 32–57, 3 < 5 *and* 2 < 7) are faster and less error-prone than comparisons of unit- decade-incompatible pairs (e.g., 37–62; 3 < 6, *but* 7 > 2). Thereby, the compatibility effect indicates influences of decision-irrelevant units on the overall comparison process. This suggests that the magnitude of units, tens, hundreds, etc. are also represented in a decomposed manner complying with the place-value structure of the Arabic number system (cf. Nuerk et al., 2011 for a review). Although the UDCE was observed for German-speakers first it is not specific for languages with inversion. It was observed in several other languages, both with inverted number words (Dutch: Ratinckx et al., 2006) and without inverted number words (English: Nuerk et al., 2005b; Moeller et al., 2009; Spanish: Macizo and Herrera, 2008; Italian: Macizo et al., 2010; Pixner et al., 2011a; Hebrew: Ganor-Stern et al., 2007, 2009). While the effect is not language-specific, it is, however, language-moderated. It was found to be more pronounced in languages with inversion both in children (Pixner et al., 2011a) and adults (Nuerk et al., 2005b). Interestingly, Pixner et al. (2011a) investigated the UDCE in German- (language with inverted number words), Italian- (without inversion) and Czech-speaking (both inverted and non-inverted number words) 1st graders. As indexed by the size of the UDCE, the interference due to decision irrelevant units was most pronounced for the language with inverted number words (German), followed by the language having both inverted and non-inverted number words (Czech) and the language without inversion (Italian).

Moreover, inversion-related language differences were also observed for the number line estimation task. Siegler and Mu (2008) showed that Chinese children's number line estimations were more accurate than those of North American children (see also Muldoon et al., 2011). Additionally, Helmreich et al. (2011) found that estimates were more accurate for Italian-speaking as compared to German-speaking children. While these language differences fit nicely with the fact that the Chinese number word system is more transparent than the English and the English more transparent than the German, one cannot exclude that the observed differences may also be driven by more general cultural differences (e.g., curricular differences). Therefore, it seems to be more promising to investigate possible influences of differences between number word systems more specifically with respect to the influence of the inversion property. In this context, Helmreich et al. (2011) identified two specific effects of inversion on children's number line estimations. First, the authors manipulated inter-digit distance of the to-be-estimated numbers [large, e.g., for 28 (8−2 = 6) vs. small, e.g., for 45 (5−4 = 1)]. Between-language differences should be more pronounced for large inter-digit distances, because marking 82 instead of 28 leads to a larger estimation error as compared to marking 45 instead of 54. And indeed, the overall advantage in estimation accuracy of Italian-speaking children was driven by target numbers with a large inter-digit distance. Second, the resulting error bias should be systematic with respect to its direction. For numbers like 49, children should overestimate the position on the number systematically because 94 (when confusing tens and units) is larger than the correct target 49 and vice versa for numbers like 51 following the same rationale. Helmreich et al. (2011) observed that this directional bias was more pronounced for German-speaking than for Italian-speaking children. Thus, even though German-speaking children refer to the same underlying mental number line representation, they were hampered to integrate tens and units into a coherent representation of a two-digit number due to the inversion property of the German number word system.

Taken together, both the results for number magnitude comparison as well as number line estimation indicate that even in non-verbal numerical tasks the transparency of the respective language's number word system influences number processing skills in children. In particular, inversion-related intransparency caused systematic and significant performance shortcomings. This raises the question whether these influences are developmentally relevant.

## Language influences on numerical development?

Generally, associations between number word inversion and numerical performance are developmentally relevant when inversion-related shortcomings predict future numerical development. Importantly, Moeller et al. (2011) were able to show such a longitudinal influence for German-speaking children. Inversion errors in transcoding and the size of the compatibility effect in 1st grade, which are both more frequent/pronounced in languages with inverted number words and indicate early place-value understanding reliably predicted arithmetic performance in 3rd grade: the more inversion transcoding errors a child committed and the larger her/his compatibility effect in 1st grade the more errors a child made in an addition task two years later. Importantly, this association was reliable even after controlling for general cognitive ability and working memory. However, they also found more specific inversion-related effects: more inversion errors in 1ist grade predicted a larger carry effect in addition as a criterion for place-value processing in 3rd grade. Finally, Moeller et al. (2011) also found that the percentage of inversion-related transcoding errors predicted the mathematics mark at the end of 3rd grade reliably: more inversion-related errors were associated with a worse mathematics mark. Importantly, this also indicates that deficiencies in early place-value understanding do not sort itself out over time.

Unfortunately, there is currently no study contrasting the influence of the inversion property on children's numerical development in a longitudinal and cross-cultural approach. However, above longitudinal influences of inversion-moderated effects for German-speaking children clearly suggest that number word structure moderates children's numerical development differentially. Fewer (or even no) inversion transcoding errors and a smaller UDCE—as also found for languages without inverted number words (see above)—were associated with better arithmetic performance.

Yet, to date the majority of research on inversion influences focuses on two-digit numbers. This seems obvious as it is the order of tens and units only that is inverted. However, one might expect inversion to also influence three-digit number processing. While in Italian or English, the neighboring number word constituents correspond to the neighboring Arabic digit, this is not the case in German: 329 is named “three-hundred-nine-and-twenty”. Thus, the neighboring number words are “three” (for hundreds) and “nine” (for units), whereas the neighboring digits are “3” (for hundreds) and “2” (for tens).

## Language effects on three-digit number processing

Currently, only few studies extend the UDCE to three-digit numbers. For English-speaking adults, Korvorst and Damian (2008) suggested that place-value and single digit magnitude information is automatically taken into account when processing three-digit numbers. Hundred-decade and hundred-unit compatibility effects (HDCE and HUCE, respectively) indicated decomposed processing of units, tens, and hundreds. However, they also observed that the HUCE (see Table 1) was smaller than the HDCE and argued that units may cause less interference than tens because of a left-to–right processing gradient for multi-digit numbers (see also Poltrock and Schwartz, 1984).

**Table 1:**
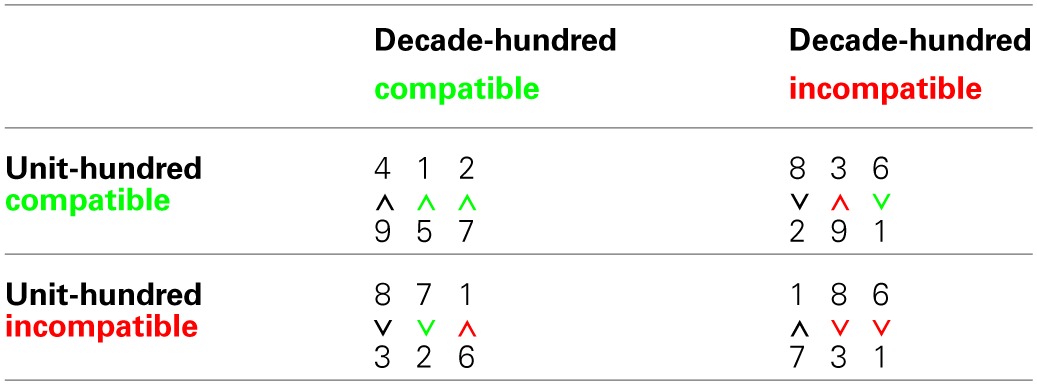
**Example stimuli**.

Place-value processing was indexed by compatibility effects (incompatible minus compatible), this means by the interference caused by the value of digits which were irrelevant for the overall magnitude decision because of their position in the digit string.

**Figure 1:**
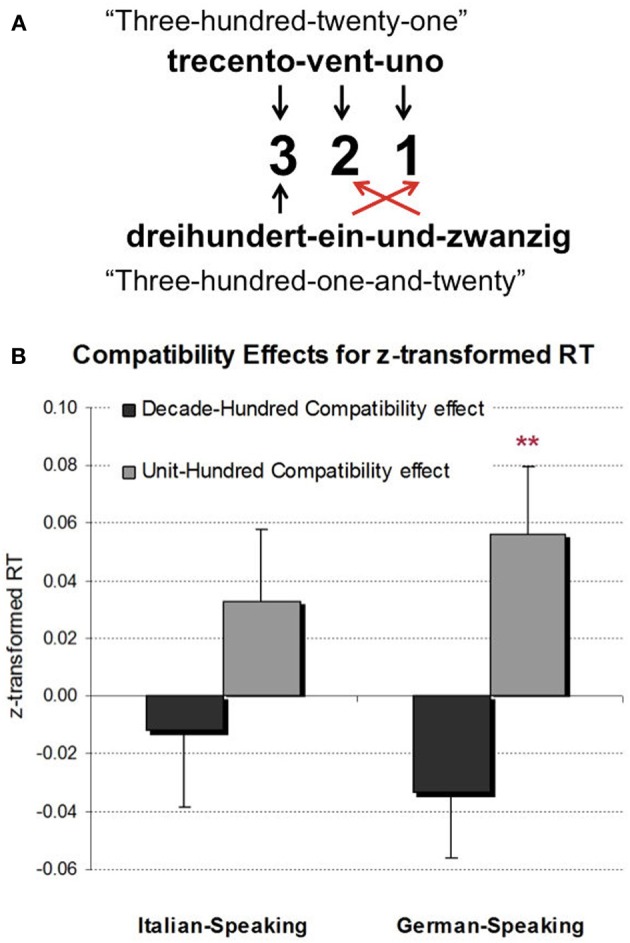
**(A)** Inversion property: The units digit is named right after the hundreds digit in German while it is named last corresponding to its position in digital notation in Italian. **(B)** Decade-hundred compatibility effects and unit-hundred compatibility effects (RT incompatible—RT compatible items in both cases) for Italian- and German-speaking 3rd graders. German-speaking 3rd graders' performance was significantly influenced by the interfering units while this was not the case for Italians. ^**^p < 0.05.

For children, Mann et al. (2012) investigated the HDCE and HUCE in German-speaking students longitudinally from grade two to four observing the HUCE to increase with age. However, the HDCE did not reach significance for any grade level. Importantly, the inversion property of the German number word system offers a plausible account for this pattern: because units are named directly after hundreds and thus before the tens (i.e., 239 → “zweihundertneununddreißig” meaning “two-hundred-nine-and-thirty,” see Figure 1A) interference due to the unit-digit might be more pronounced than interference due to the tens-digit. Thus, interference by the neighboring number word constituents was more pronounced than by the neighboring Arabic digits.

However, because there are only these data from German-speaking children, conclusions about possible language differences have to be drawn cautiously as a direct contrast of compatibility effects for three-digit numbers between different language groups is still missing. To present a first perspective on language differences for numbers beyond the two-digit number range, we briefly present additional new data on three-digit number comparison of 82 Italian-speaking 3rd graders (40 female; mean age 9;0 years; SD = 3.5 months; non-inverted number words) to contrast them with those of the German sample of Mann et al. (2012; 96 children, 47 female, mean age 9;4 years, SD = 4.4 months; inverted number words).

All participants completed a three-digit number magnitude comparison task. In the stimulus set of 80 between-hundred three-digit number pairs the factors decade-hundred compatibility and unit-hundred compatibility were manipulated orthogonally (see Table 1). Children had to indicate the larger of two simultaneously presented numbers by pressing a corresponding button. RT analyses were based exclusively on correct between-decade trials. Additionally, a trimming procedure first eliminated RT shorter than 200 ms and larger than 8000 ms and then all RT deviating from the individual's mean by more than 3 SD. As RT means and SD varied considerably between participants, RT were z-transformed (zRT) prior to the analyses. Please note, the pattern for error rates was similar (*r* = 0.80) but due to the generally low error rates (*M* = 4.0%; *SD* = 2.4%) less discriminating.

We observed a regular HUCE [*F*_(1, 176)_ = 6.77, *p* < 0.05] indicating that hundred-unit compatible items were responded to faster (1431 ms) than hundred-unit incompatible items (1450 ms). However, as the pattern of compatibility effects was similar in both language groups the interaction of language group and compatibility was not significant [*F*_(1, 176)_ = 3.56, *p* = 0.56]. Nevertheless, as we had a specific hypothesis regarding the presence of the compatibility effect, we inspected the simple effects. These indicated that the HUCE was only significant for German-speaking 3rd graders [*t*_(95)_ = 2.50, *p* < 0.05, see Figure 1B] with hundred-unit compatible items responded to faster (1258 ms) than hundred-unit incompatible items (1278 ms), but not for Italian-speaking 3rd graders [*t*_(81)_ = 1.26, *p* = 0.21].

These findings further corroborate the assumption that magnitude processing of multi-digit numbers and, in particular, the processing of place-value information is moderated by linguistic characteristics such as the inversion property of the respective number word structure. Unit interference was only significant for German-speaking 3rd graders. As previously found for two-digit numbers, these results suggest that proximity not only in digital but also in verbal number word notation is a relevant predictor for place-value compatibility effects. On a more general level, the data provide further support to the notion that language impacts on cognitive (number) processes, even those supposed to be non-verbal. Interestingly, the pattern of compatibility effects was similar for both languages. This fits nicely with the results of Helmreich et al. (2011, see above). In a number line estimation task, both estimation errors due to large inter-digit distances as well as the directional bias of estimation errors were more pronounced for German-speaking but nevertheless present for Italian-speaking children. However, while this influence of inversion on number line estimation was significant in direct comparisons (see also Nuerk et al., 2005a,b; Pixner et al., 2011a for evidence on magnitude comparison), it was not significant in the data presented here. Nevertheless, the simple effect analyses indicate that three-digit number processing also seems to be influenced by inversion—although to a weaker degree compared to two-digit numbers. Moreover, this suggests that other language invariant aspects moderate (multi-digit) number processing. As regards three-digit numbers, perceptual attributes such as lateral masking effects may be an influencing factor because hundred and unit digits flank the tens digit in the center from both sides, possibly overcoming (linguistic) effects for those digits.

## Conclusion and perspectives

The current review indicates that numerical development may not be language-specific but is, however, moderated by language. Language influences in children's numerical development seem to be more pronounced for multi-digit numbers complying with the fact that differences between number word systems studied so far have been stronger for multi- than for single-digit numbers. In particular, place-value integration is more difficult in languages with inverted number words in which units are named before tens. New data comparing German-speaking and Italian-speaking children in three-digit number comparison generalize this assumption beyond the two-digit number range. The findings discussed in this review are highly relevant for numerical development since inversion-related difficulties were shown to predict later arithmetic performance (Moeller et al., 2011).

However, apart from specificities of number word systems there are other more general language specificities which may influence number processing such as, for instance, reading direction (e.g., Shaki et al., 2009, see Göbel et al., 2011 for a review). These studies provide conclusive evidence that reading direction influences number processing in adults, in particular spatial numerical associations. Shaki et al. (2009) observed that English-speaking participants (who read both words and numbers from left to right) systematically associated small numbers with left and large numbers with right whereas this association was reversed for Palestinians (reading words and Arabic-Indic numbers from right to left). Against this background, it might be interesting to further investigate language influences by means of different number systems as well as reading direction to further evaluate the impact of language on numerical development.

Another interesting question regards the directionality of influences between number processing and language. To the best of our knowledge there is currently no study investigating possible influences of number processing on language. One possible reason for this uni-directional research bias might be that for an approach paralleling the one pursued in most of the studies described above one would need two cultures speaking the same language but having a different number system to investigate the influence of number processing on language. Unfortunately, we do not know any such case. Maybe it might be possible to address this issue in a less strict manner in languages such as Czech (with both inverted and non-inverted number words). So far, there is only evidence for specific difficulties in the number domain associated with the use of the inverted form (e.g., Pixner et al., 2011a,b). However, one might think of investigating how the use of either number word system influences language processing in these children in future studies.

### Conflict of interest statement

The authors declare that the research was conducted in the absence of any commercial or financial relationships that could be construed as a potential conflict of interest.
